# The Impact of Climate Change on Infectious Disease Transmission: Perceptions of CDC Health Professionals in Shanxi Province, China

**DOI:** 10.1371/journal.pone.0109476

**Published:** 2014-10-06

**Authors:** Junni Wei, Alana Hansen, Ying Zhang, Hong Li, Qiyong Liu, Yehuan Sun, Shulian Xue, Shufang Zhao, Peng Bi

**Affiliations:** 1 Department of Epidemiology, School of Public Health, Shanxi Medical University, Taiyuan, Shanxi, China; 2 Discipline of Public Health, School of Population Health, The University of Adelaide, Adelaide, Australia; 3 Sydney School of Public Health, The University of Sydney, Sydney, NSW, Australia; 4 Shanxi Center for Disease Control and Prevention, Taiyuan, Shanxi, China; 5 State Key Laboratory for Infectious Diseases Prevention and Control, National Institute for Communicable Disease Control and Prevention, Chinese Center for Disease Control and Prevention, Beijing, China; 6 Shandong University Climate Change and Health Center, Jinan, Shandong, China; 7 Department of Epidemiology and Biostatistics, School of Public Health, Anhui Medical University, Hefei, Anhui, China; University of Texas Medical Branch, United States of America

## Abstract

There have been increasing concerns about the challenge of emerging and re-emerging infectious diseases due to climate change, especially in developing countries including China. Health professionals play a significant role in the battle to control and prevent infectious diseases. This study therefore aims to investigate the perceptions and attitudes of health professionals at the Centers for Disease Control and Prevention (CDC) in different levels in China, and to consider adaptation measures to deal with the challenge of climate change. In 2013, a cross-sectional questionnaire survey was undertaken among 314 staff in CDCs in Shanxi Province, China, whose routine work involves disease control and prevention. Data were analyzed using descriptive methods and logistic regression. A majority of the CDC staff were aware of the health risks from climate change, especially its impacts on infectious disease transmission in their jurisdictions, and believed climate change might bring about both temporal and spatial change in transmission patterns. It was thought that adaptation measures should be established including: strengthening/improving currently existing disease surveillance systems and vector monitoring; building CDC capacity in terms of infrastructure and in-house health professional training; development and refinement of relevant legislation, policies and guidelines; better coordination among various government departments; the involvement of the community in infectious disease interventions; and collaborative research with other institutions. This study provides a snapshot of the understanding of CDC staff regarding climate change risks relevant to infectious diseases and adaptation in China. Results may help inform future efforts to develop adaptation measures to minimize infectious disease risks due to climate change.

## Introduction

Many studies have reported the impact of climate variability on the transmission of infectious diseases, including vector-borne, rodent-borne, food-borne and water-borne diseases [1]–[5], indicating that increasing temperature and changes in patterns of rainfall will continue to affect the transmission of infectious diseases, temporally and spatially [3], [6]–[8]. The emerging and re-emerging of climate-sensitive infectious diseases will pose a great challenge to our already overloaded healthcare system worldwide. Although there are considerable policies and public health interventions to reduce the health burden of infectious diseases, current strategies, policies, guidelines and measures are not specifically designed to meet the challenge of climate sensitive infectious diseases. Environmental, climatic and demographic changes, as well as increasing urbanization, may have a considerable effect on the transmission of infectious diseases, particularly in developing countries including China given their relatively lower socioeconomic status (SES), and under-developed healthcare system and workforce [9], [10].

Similar to other countries, China has experienced climate variation and fluctuation, as well as extreme weather events in the past 50 years, which may have had a considerable effect on the frequency and distribution of infectious diseases [7], [11]. Although China has been aware of the urgency to address climate change and its health impacts, strategies and measures specifically responding to climate-related infectious diseases are very limited [12], [13].

The Centers for Disease Control and Prevention (CDC) in China are governmental public health agencies whose main goal is to protect public health and safety through the control and prevention of disease. There are three levels of CDCs in each Province: (1) a provincial level CDC whose main duties are to provide professional management, technical assistance and organizational coordination to the CDCs in lower levels; (2) prefecture-level city CDCs, and (3) district/county CDCs which are responsible for infectious disease detection, outbreak investigation, response, control and elimination. In order to reduce the adverse consequences of climate change on infectious diseases, it is useful to better understand the current knowledge and perceptions of CDC staff regarding impacts of climate change on infectious diseases and relevant response measures. For this reason, we conducted a representative survey of staff from the three levels of CDCs in Shanxi Province. The results may aid in understanding the current knowledge and attitude of CDC professionals about the relationship between climate change and infectious diseases; identify the gaps in policy and practice in terms of infectious disease response due to climate change; and provide suggestions for strategies and measures to deal with the impacts of climate change on infectious diseases.

## Materials and Methods

### Ethics statement

The study was approved by the Ethics Committee of Shanxi Medical University (No. 2013092), China, and conducted in accordance with its guidelines. Verbal and written informed consent were obtained from every participant enrolled in this study. In order to collect the survey data in an objective way, verbal consent to participate by telephone was firstly obtained prior to the investigation. Subsequently, written informed consent was obtained by every respondent signing the final page of the questionnaire where indicated. This consent procedure was approved by the Ethics Committee of Shanxi Medical University.

### Background information

Located in North China, Shanxi Province has a total area of 156,000 sq km with and a population of 36.11 million. Shanxi lies between latitude 34°34'-40°44' north and longitude 110°15'-114°32' east. It has a temperate, continental, monsoonal climate with four distinct seasons. The average temperature in January is in the range −16°C to −2°C and in July between 19°C and 28°C. Average annual rainfall is between 350 to 700 mm. Shanxi has 11 prefecture-level divisions, which are subdivided into 23 districts, 11 county-level cities, and 85 counties. There is a CDC in each county, district and city, in addition to the Shanxi Provincial CDC.

### Data collection

The targeted study population was the health professionals who were employees of CDCs at the time of the study in Shanxi Province. The participating CDCs were drawn from the north, central and south regions of the Province.

A questionnaire was developed after a review of the literature on knowledge, attitude and strategies in relation to climate change. The questionnaire included demographic information, perceived knowledge towards potential health impacts of climate change, daily work and responses regarding infectious diseases, perceptions regarding infectious diseases in the context of global climate change, and measures to deal with future challenges due to climate change. All questions had close-ended responses. The draft questionnaire was validated by experts and piloted among a selection of 15 staff in Shanxi Provincial CDC. Relevant revisions were made after the pilot survey. Two well-trained research associates collected the data using the questionnaire. In order to maximise response rates, the investigators firstly made a telephone call to health professionals working in infectious disease control and prevention and other relevant branches in the selected CDCs, and invited them to participate in the survey, briefly introduced the survey's aim and main content to them, then asked about their willingness to participate. Of the 350 staff being approached, 330 expressed an interest in participating in the study. After verbal consent was obtained from respondents, questionnaires were mailed out in early September 2013, and participants were requested to return their completed questionnaire using a supplied reply-paid envelope before the end of the month. A total of 330 questionnaires were distributed, and 314 were returned from sixteen CDCs (including 1 provincial CDC, 8 prefecture-level CDCs and 7 district and county CDCs), with a response rate of 95.2%. The investigators checked all the questionnaires and no data were missing.

### Statistical analysis

Data were entered into Epidata version 3.1 (http://www.epidata.dk.) database and imported into SPSS version 16.0 (SPSS Inc. Chicago, USA) for statistical analysis. Descriptive statistics were used to illustrate respondents' demographic characteristics and percentages of categorical variables. Chi square or Fisher's exact tests (expected cell frequencies less than or equal to five) were used to test for the relationships between demographic variables and perception variables. Where responses were ordinal, multivariate ordinal logistic regression analysis was used to explore the association with demographic variables, and the odds ratios (*OR*) with 95% confidence intervals (*CI*) were calculated.

## Results

### Respondents' demographic characteristics

Table 1 shows the demographic characteristics of the 314 respondents (ages 20–60 years). More than half of the respondents (60.5%) were female. Respondents were from the provincial CDC (18.8%), prefecture-level city CDCs (66.9%) and district/county level CDCs (14.3%), respectively. In terms of level of education, a majority of the respondents (69.7%) had an undergraduate degree level or above. Fifty-one percent of respondents had been employed at the CDC less than 9 years. There were 40.4% employed at both junior and intermediate level and 32.8% were managers. The professional specialties of respondents were disease control (including disease surveillance, sterilization, immunization, health education) (78.9%), public health (including environmental, occupational, children's health, epidemiology and food hygiene) (16.6%), medical laboratory (32.3%) and emergency response and management (14.1%).

**Table 1:** Demographic characteristics of the participants (N = 314).

Characteristics		Number	Percent (%)
**Age group (years)**			
	20–39	146	46.5
	≥40	168	53.5
**Gender**			
	Male	124	39.5
	Female	190	60.5
**Educational Level**			
	Below undergraduate	95	30.3
	At or above undergraduate level	219	69.7
**Levels of CDC**			
	Provincial	59	18.8
	Prefecture-level city	210	66.9
	District/county	45	14.3
**Length of employment at CDC (years)**			
	≤9	160	51.0
	10–19	79	25.2
	20–39	75	23.9
**Professional level**			
	Junior	127	40.4
	Intermediate	127	40.4
	Senior	60	19.1
**Unit Manager**			
	No	211	67.2
	Yes	103	32.8
**Specialty (multiple response)** a			
	Infectious Disease control	247	78.9
	Public health	52	16.6
	Medical laboratory	101	32.3
	Emergency response and management	44	14.1

a Multiple response was analyzed by using multiple dichotomy method to form a multiple response set; percent of cases column is the percentage of valid cases represented by each category, and these percentages will sum to more than 100% if at least one person made more than one response.

### Respondents' perceived knowledge towards potential health impacts of climate change

Respondents were asked how they perceived the vulnerable populations due to climate change (Table 2). The majority (81.5%) indicated the elderly were vulnerable to climate change/extreme weather, followed by infants and children (68.2%), people with existing diseases (65.9%), outdoor workers (62.4%) and people with lower SES (19.4%). Respondents were then asked their perception about impacts of climate change on infectious diseases (Table 2). A majority of respondents (70.4%) believed that infectious diseases were sensitive to climate change/extreme weather. Moreover, 77.7% of respondents indicated global warming would aggravate the burden of vector-borne diseases, followed by air-borne diseases (69.7%), water-borne diseases (67.8%), and food-borne diseases (49.7%). Almost half the respondents believed that climate change/extreme weather was a driving force for the resurgence of old infectious diseases and the emerging of new infectious diseases. Although 75.8% of respondents thought the impact of climate change on infectious disease transmission was a public health problem, only 8.6% of them considered the challenge to be serious and conducted related research in their daily work.

**Table 2:** CDC staff's perception about the health impacts from climate change (N = 314).

Potential health impacts of climate change		Number	Percent (%)
**Which population do you think is at the most risk from climate change/extreme weather?** *			
	Infants and children	214	68.2
	Young adults	13	4.1
	Middle-aged	22	7.0
	The elderly	256	81.5
	Lower SES	61	19.4
	Outdoor workers	196	62.4
	People with existing diseases	207	65.9
	Others	4	1.3
**What diseases do you think are sensitive to climate change/extreme weather?** *			
	Respiratory diseases (e.g. asthma, pneumonia, chronic block pulmonary emphysema)	249	79.3
	Cardiovascular disease (e.g. hypertension, heart disease)	253	80.3
	Urinary system diseases (e.g. nephritis, kidney stones)	35	11.1
	Digestive System diseases (e.g. gastritis, hepatitis)	116	36.9
	Infectious diseases	221	70.4
	Others	5	1.6
**Do you think global warming will aggravate the transmission of these diseases?** *			
	Air-borne diseases	219	69.7
	Water-borne diseases	213	67.8
	Directly contact transmitted diseases	64	20.4
	Food-borne diseases	156	49.7
	Vector-borne diseases	244	77.7
	Soil-borne diseases	93	29.6
	Rodent-borne diseases	42	13.4
	Vertical transmitted diseases	25	8.0
**Is climate change/extreme weather the reason for emerging and re-emerging infectious diseases?**			
	Extremely likely	72	22.9
	Very likely	145	46.2
	Somewhat likely	87	27.7
	Less likely	10	3.2
**Do you think global warming trends will lead to higher incidence and wider geographic range of vector-borne diseases (malaria, dengue and Japanese encephalitis), rodent-borne diseases (hemorrhagic fever), water-borne diseases and food-borne diseases (dysentery, schistosomiasis, cholera)?**			
	Extremely likely	91	29.0
	Very likely	150	47.8
	Somewhat likely	63	20.1
	Less likely	10	3.2
**Have you considered the impact of climate change on infectious diseases in your work?**			
	Considered and conducted related researches	27	8.6
	Just think about	238	75.8
	Not at all considered	49	15.6

*Percentage total may add up to more than 100% as multiple responses were permissible.

There was a significant association between the length of employment at CDC (χ^2^ = 12.094, *p* = 0.002), working in the areas of emergency response (*p* = 0.027) and concerns about the impact of climate change on infectious disease. Staff who had been employed between 20 and 39 years (90.7%) were more likely to believe that global warming would increase the incidence of vector-borne disease. Staff performing duties for emergency response (47.7%) were more likely to believe climate change would increase incidence and expand epidemic regions of infectious diseases.

### Respondents' perceptions of current existing infectious disease surveillance and epidemic responses

In terms of the efficiency of the current infectious disease surveillance system in China, 79.7% of CDC staff believed it was excellent, 21.7% thought it worked “Extremely well”, and 58% said “Very well”. For the capacity of the system to respond to epidemics of infectious diseases, 58.6% indicated responses were “Extremely timely” for air-borne diseases, food-borne diseases (51.9%), water-borne diseases (47.5%) and vector-borne diseases (42.7%). There was a statistically significant association between CDC level (*p* = 0.001), professional level (*p* = 0.020), and assessment for surveillance of infectious disease. Staff from prefecture-level city CDCs (59.0%) and district/county CDCs (66.7%) were more likely to believe that the surveillance of infectious diseases is performed “very well” than those from the provincial CDC (47.5%). Lower professional level respondents were more confident about the surveillance system than senior staff.

### Perceptions towards coping with infectious disease in the context of global climate change

A number of respondents believed that global warming would lead to higher incidence and wider geographic range of vector-borne diseases, rodent-borne diseases, water-borne and food-borne diseases, such as malaria, dengue, Japanese encephalitis, hemorrhagic fever with renal syndrome, dysentery and cholera, with 29.0% believing it was “Extremely likely”, and 47.8% believed it was “Very likely” (Table 2).

Most respondents (80.9%) believed public health professionals and local CDCs should take effective strategies and measures to deal with the health challenges posed by climate change. More than half of respondents (55.7%) believed meteorological impacts should be considered in current prevention and control regulations for infectious diseases in local CDCs.

Table 3 describes respondents' perception towards response measures of surveillance and scientific research. More than 90% of respondents believed it was either extremely important or very important to strengthen surveillance and improve quality of data of infectious diseases, followed by vector surveillance (86.3%), vulnerable groups monitoring and protection (74.5%), meteorological factor monitoring (68.2%), and clinical monitoring of patients (63.6%). In terms of how CDC could improve its capability to meet the potential increasing burden of infectious diseases due to climate change, respondents (81.3%) believed it was important to increase investment in scientific research, followed by assessing the risk of spreading infectious diseases due to climate change (80.3%), improving emergency response mechanisms for the disease outbreak (79.6%), enhancing surveillance and projection capabilities (74.2%), and identifying high risk climatic zones (68.4%).

**Table 3:** CDC staff's perceptions towards infectious disease surveillance and scientific research of response measures (N = 314).

Response measures to climate change		EI	VI	JS	UI
		n (%)	n (%)	n (%)	n (%)
**How important do you think these response measures are in terms of dealing with the threat of infectious diseases due to climate change?**					
	Improve the quality of disease surveillance data	175 (55.7)	113 (36.0)	21 (6.7)	5 (1.6)
	Strengthen the surveillance of infectious diseases, especially vector-borne diseases, waterborne and food-borne disease	181 (57.6)	123 (39.2)	8 (2.6)	2 (0.6)
	Vector surveillance (such as mosquitoes and other insects)	115 (36.6)	156 (49.7)	41 (13.1)	2 (0.6)
	Meteorological variable observation	81 (25.8)	133 (42.4)	86 (27.3)	14 (4.5)
	Vector breeding site surveillance	96 (30.6)	157 (50.0)	51 (16.2)	10 (3.2)
	Vulnerable groups surveillance and protection	80 (25.5)	154 (49.0)	63 (20.1)	17 (5.4)
	Clinical monitoring of patients	62 (19.7)	138 (43.9)	97 (31.0)	17 (5.4)
**How important are these aspects of scientific research in terms of dealing with the health impacts of climate change?**					
	Enhancing surveillance and projection capacities	77 (24.5)	156 (49.7)	70 (22.3)	11 (3.5)
	Assessing the risk of spreading infectious diseases due to climate change	92 (29.3)	160 (51.0)	52 (16.5)	10 (3.2)
	Identifying high risk climatic zones	77 (24.5)	138 (43.9)	83 (26.5)	16 (5.1)
	Improving emergency response mechanisms for disease outbreaks	105 (33.4)	145 (46.2)	47 (15.0)	17 (5.4)
	Increasing investment in scientific research associated with addressing climate change	133 (42.4)	122 (38.9)	48 (15.2)	11 (3.5)

Note: EI  =  Extremely important; VI  =  Very important; JS  =  Just so so; UI  =  Unimportant.

Table 4 describes respondents' perceptions towards capacity building, legislation and health intervention measures in terms of dealing with the challenges of infectious diseases due to climate change. A large majority of respondents believed that these adaptation measures were either extremely important or very important. Infrastructure (86.3%), staff in-house training (85.3%), health education (84.7%) and information sharing (80.5%) were believed to be important to maintain the capacity to effectively control and prevent infectious diseases within the context of climate change in China. Respondents (83.4%) thought that it was also extremely or very important to coordinate the contributions from different government departments in the climate change adaptation policy-making process (83.4%), followed by policies, legislation and regulations to address climate change (72.3%). In terms of infectious disease interventions, respondents (88.5%) believed it was important to control vector breeding sites for vector-borne diseases, improve drinking water and sanitation (85.1%), and promote individual protection (84.7%) and food safety (83.5%).

**Table 4:** CDC staff's perceptions towards capacity building, legislation and health intervention measures of infectious diseases (N = 314).

Response measures to climate change		EI	VI	JS	UI
		n (%)	n (%)	n (%)	n (%)
**How important are these disease control and prevention ability construction to adapt to climate change?**					
	Infrastructure development/refinement (e.g. improve disease surveillance platform, online disease notification system)	142 (45.2)	129 (41.1)	39 (12.4)	4 (1.3)
	Staff in-house training	114 (36.3)	154 (49.0)	40 (12.7)	6 (1.9)
	Cross department information sharing	105 (33.4)	148 (47.1)	53 (16.9)	8 (2.5)
	Community Health education	103 (32.8)	163 (51.9)	43 (13.7)	5 (1.6)
**Policies, legislation and regulations formulation to address climate change**		102 (32.5)	125 (39.8)	73 (23.2)	14 (4.5)
**Decision-making coordination among government departments**		116 (36.9)	146 (46.5)	41 (13.1)	11 (3.5)
**Infectious Disease prevention**					
	Improve living conditions	104 (33.1)	147 (46.8)	53 (16.9)	10 (3.2)
	Individual protection	110 (35.0)	156 (49.7)	38 (12.1)	10 (3.2)
	Food safety	118 (37.6)	144 (45.9)	43 (13.7)	9 (2.9)
	Control the environment of vector breeding sites	124 (39.5)	154 (49.0)	29 (9.2)	7 (2.2)
	Improve drinking water and sanitation	122 (38.9)	145 (46.2)	36 (11.5)	11 (3.5)
	Insecticide use, mosquito control, deratization, etc.	141 (44.9)	126 (40.1)	30 (9.6)	17 (5.4)

Note: EI  =  Extremely important; VI  =  Very important; JS  =  Just so so; UI  =  Unimportant.

In terms of adaptation against the health impacts of climate change in the future, respondents (82.5%) believed that prevention was an important adaptation measure to control infectious diseases, followed by information sharing (81.8%), drinking water safety (67.5%), timely and effective coordination of health action in an emergency (65.6%) and international cooperation (61.8%) (Table 5).

**Table 5:** CDC staff's perceptions towards future strategies and measures (N = 314).

How important are these strategies and measures?	EI	VI	JS	UI	NA
	n (%)	n (%)	n (%)	N (%)	n (%)
Prevention first	214 (68.2)	45 (14.3)	14 (4.5)	7 (2.2)	34 (10.8)
Strengthen international cooperation	101 (32.2)	93 (29.6)	47 (15.0)	18 (5.7)	55 (17.5)
Establish a global infectious disease monitoring and response system for information sharing	163 (51.9)	94 (29.9)	24 (7.6)	5 (1.6)	28 (8.9)
Drinking water safety	92 (29.3)	120 (38.2)	37 (11.8)	12 (3.8)	53 (16.9)
Timely and effectively coordinate health action in an emergency event	71 (22.6)	135 (43.0)	37 (11.8)	10 (3.2)	61 (19.4)
Provide high space-time resolution of data which could be incorporated into GIS in future	37 (11.8)	103 (32.8)	88 (28.0)	15 (4.8)	71 (22.6)
Promote adaptation actions through in-house training and legislation	28 (8.9)	79 (25.2)	76 (24.2)	44 (14.0)	87 (27.7)
Behavior change and medical intervention	24 (7.6)	48 (15.3)	85 (27.1)	65 (20.7)	92 (29.3)

Note: EI  =  Extremely important; VI  =  Very important; JS  =  Just so so; UI  =  Unimportant; NA  =  No answer was given.

The multivariate ordinal logistic regression analysis was used to examine the associations between adaptation measures (dependent variables) and demographic characteristics (independent variables). Results showed that when undergraduate education level or above was compared with below undergraduate, respondents with higher education level were more likely to believe that strengthening surveillance (OR = 1.900, 95%CI: 1.022–3.532, *p* = 0.042) and decision-making coordination (OR = 1.802, 95%CI: 1.021–3.180, *p* = 0.042) were important adaptation measures. Respondents who performed duties for emergency response were more likely to believe that strengthening surveillance (OR = 4.415, 95%CI: 1.682–11.557, *p* = 0.003) and vector monitoring (OR = 3.916, 95%CI: 1.751–8.767, *p* = 0.001) were important adaptation measures than non-emergency professionals. Respondents from the provincial CDC were more likely to believe that vector surveillance (OR = 2.710, 95%CI: 1.119–6.303, *p* = 0.027) was an important adaptation measure than those from district/county CDCs. Compared with non-managers, managers were more likely to believe that decision-making coordination was an important adaptation measure to control and prevent infectious diseases (OR = 1.826, OR95%CI: 1.082–3.080, *p* = 0.024) (Table 6).

**Table 6:** Multivariate ordinal logistic regression between demographic variables and measures related to climate change.

Dependent variables	Independent variables	*OR*	95% *CI*	*p* value
Strengthen the surveillance of infectious diseases	Educational level (At or above undergraduate/Below undergraduate)	1.900	1.022–3.532	0.042
	Emergency response and management (Yes/No)	4.415	1.682–11.577	0.003
Vector surveillance	Levels of CDC (Provincial/District and county)	2.710	1.119–6.303	0.027
	Emergency response and management (Yes/No)	3.916	1.751–8.767	0.001
Decision-making coordination among government departments	Educational level (At or above undergraduate/Below undergraduate)	1.802	1.021–3.180	0.042
	Unit Manager (Yes/No)	1.826	1.082–3.080	0.024

## Discussion

Given the potential health impacts due to climate change, it is important and necessary for the healthcare system to prepare for the challenge, both at present and for the future. As government organizations, CDCs supply the services that underpin all aspects of population health including infectious diseases control and prevention. Therefore, it is useful to understand the attitude and perception of CDC staff on climate change and infectious disease control, to identify knowledge and policy/guideline gaps and to explore current and future adaptation measures. For the first time, this study assesses these issues among the health professionals from CDCs in China. Results may provide useful evidence for policy-makers and service providers in the development of climate change policies.

Vulnerable populations may be more sensitive to climate-related infectious diseases and the results of this study showed that the CDC staff believed that people most vulnerable to extreme weather and climate change were the elderly, followed by infants and children, people with existing diseases and outdoor workers. This seems consistent with findings from other studies on susceptible populations to climate change [14]–[23]. In addition, previous studies showed that people of lower SES were also vulnerable to climate change [18], [24], [25]. In this study, however, only 19.4% of respondents thought that the poor had a greater health risk related to climate change, indicating that more updated knowledge and in-house training should be provided to the health professionals within CDC.

The majority (70.4%) thought infectious diseases were sensitive to climate change/extreme weather, although surprisingly, this ranked third behind cardiovascular disease and respiratory disease. This indicates almost 30% either do not believe there will be an impact on infectious diseases; or are reluctant to assign climate sensitivity to all infectious diseases. Generally studies have found a likely association between climate variability and vector-borne, rodent-borne, food-borne or waterborne diseases only [1], [26]–[29]. Notwithstanding, more than two thirds of respondents thought that global warming trends would lead to higher incidence and wider geographic range of vector/rodent-borne and food-borne diseases, such as dengue fever, malaria, Japanese encephalitis, hemorrhagic fever, dysentery, schistosomiasis and cholera. These indicate that more attention, investment and resource should be provided to vector-borne, rodent-borne, water-borne and food-borne diseases in future in the context of climate change. It is also noted that only one third of staff in the CDC have a good understanding of the impact of climate change on the transmission of infectious diseases, suggesting in house training is necessary to provide updated knowledge to CDC health professionals to maintain the necessary capacity to meet the future challenge. In addition, this study found respondents with long employment with CDC (20–39 years) and those who work in the area of emergency response were more likely to believe global warming would affect the incidence of vector-borne disease and expand the epidemic region of infectious diseases. This could be explained by their long service experience and emergency response ability. The results also imply that regular workshops need to be provided for the CDC staff in other areas.

Less than 80% of respondents indicated that the surveillance of infectious diseases was excellent in their jurisdictions. When coping with epidemics of infectious diseases, they felt responses were “Extremely timely” regarding air-borne disease (58.6%), food-borne disease (51.9%), water-borne disease (47.5%) and vector-borne disease (42.7%). The results show that although almost 80% of staff trust the surveillance system, there may still be some deficiency in the surveillance and response system to infectious diseases, including the possible delay of surveillance data submission from lower level CDCs to upper level CDCs. The staff who were from prefecture-level city and district/county CDC were more likely to believe response was “Very timely” on the surveillance of infectious disease than those from provincial CDC, and junior staff were more likely to believe “Very timely” on the surveillance than senior professionals.

Although the influence of climate change on infectious disease continues to be elucidated, much work is still required in terms of adaptation measures for climate-related infectious disease. The World Health Assembly passed an important resolution calling on the health community to protect health from climate change [30]. Adaptation to infectious disease impacts of climate change will be an evolving process in local health departments. We found most respondents believed public health professionals and local CDCs should take effective strategies and measures to cope with the health impacts of climate change, and more than half of respondents believed meteorological impacts (such as variation in temperature and rainfall) should also be considered in infectious disease control and prevention practice in China.

The fifth assessment report of the Intergovernmental Panel on Climate Change (IPCC AR5) pointed out that impacts and health risks related to climate change can be reduced and managed through adaptation measures [31], [32]. Many approaches for adaptation are appropriate for local initiatives. In this study, respondents' perception towards adaptation measures included: 1) strengthening surveillance and vector monitoring; 2) scientific research; 3) improving CDC's capacity to adapt to climate change; 4) the establishment of relevant policies, legislation and regulations; 5) coordination of various government departments for decision-making; and 6) disease intervention. A majority of respondents believed these adaptation measures were important to reduce the adverse impacts of climate change on the control of infectious diseases. These findings extend, and are largely consistent with, two literature review studies [7], [33]. Moreover, our findings show that the CDC staff in China with higher education levels and responsible for emergency management work are more likely to place importance on increasing surveillance capacity; staff in the provincial CDC and those working in emergency response units support improvement in vector surveillance; and senior staff think more coordination during the decision-making process is important. The results may imply better adaptation measures need to be employed to address climate-related infectious diseases.

In addition, respondents' perceptions towards adaptation measures in the future were also explored. Most respondents believed that prevention, information sharing, drinking water safety, coordinating health action in an emergency situation, and international cooperation were important adaptation measures.

There are limitations in this study. First, this study was conducted in one province and therefore results are not representative of all CDCs across China, given its wide geographic areas and large population. Second, perceptions towards mitigation measures (e.g., reducing emissions of greenhouse gas) were not included in this survey. Third, there are some ‘double’ questions with a single response (e.g. behavior change and medical intervention; climate change/extreme weather). Additionally, the survey was administered in Chinese and there may be an occasional mismatch of terms in the English translation. Despite these limitations, these findings are useful as they provide information regarding perceptions of CDC staff towards the impacts of climate change in terms of climate-related infectious diseases. Such information may also be useful for health departments in formulating effective adaptation plans to reduce the adverse effect of climate change on infectious disease transmission. Further research is needed to examine attitudes towards mitigation measures and how CDCs can play an active and effective role in climate adaptation and mitigation.

## Supporting Information

Table S1 **The length of repondents' employment at CDC and their concerns about the impact of climate change on vector-borne disease.**
(DOCX)

Table S2 **The views of emergency response and management staff on expanding epidemics of infectious diseases.**
(DOCX)

Tables S3 **The significant association between different levels of CDC staff and their perception towards infectious disease surveillance.**
(DOCX)

Table S4 **Local CDC staff's perceptions towards the response to infectious disease epidemics (N = 314).**
(DOCX)
