# Supplementary material for: The Impact of Climate Change on Infectious Disease Transmission: Perceptions of CDC Health Professionals in Shanxi Province, China
Source: PLoS One. 2014 Oct 6;9(10):e109476. doi: 10.1371/journal.pone.0109476 (PMC4186885; doi:10.1371/journal.pone.0109476)
Supplement: Table S1 — The length of repondents' employment at CDC and their concerns about the impact of climate change on vector-borne disease. (DOCX) [file pone.0109476.s001.docx]

**Table S1.** The length of repondents’ employment at CDC and their concerns about the impact of climate change on vector-borne disease

| **Length of employment at CDC (years)** | **Impacts of climate change on vector-borne disease** | | | **χ^2^** | ***p*** |
| --- | --- | --- | --- | --- | --- |
|  | **Yes** | **No** | **Total** |  |  |
| ≤9 | 113 | 47 | 160 |  |  |
| 10-19 | 63 | 16 | 79 | 12.094 | 0.002 |
| 20-39 | 68 | 7 | 75 |  |  |
| Total | 244 | 70 | 314 |  |  |
