# Supplementary material for: The Impact of Climate Change on Infectious Disease Transmission: Perceptions of CDC Health Professionals in Shanxi Province, China
Source: PLoS One. 2014 Oct 6;9(10):e109476. doi: 10.1371/journal.pone.0109476 (PMC4186885; doi:10.1371/journal.pone.0109476)
Supplement: Table S2 — The views of emergency response and management staff on expanding epidemics of infectious diseases. (DOCX) [file pone.0109476.s002.docx]

**Table S2.** The views of emergency response and management staff on expanding epidemics of infectious diseases

| **Emergency response and management staff** | **Expanding the epidemic of infectious diseases** | | | | | **χ^2^** | ***p*** |
| --- | --- | --- | --- | --- | --- | --- | --- |
|  | **EL** | **VL** | **SL** | **LL** | **Total** |  |  |
| Yes | 21 | 17 | 5 | 1 | 44 |  |  |
| No | 70 | 133 | 58 | 9 | 270 | 9.163 | 0.027 |
| Total | 91 | 150 | 63 | 10 | 314 |  |  |

Note: EL= Extremely likely; VL = Very likely; SL = Somewhat likely; LL = Less likely.
