# Supplementary material for: The Impact of Climate Change on Infectious Disease Transmission: Perceptions of CDC Health Professionals in Shanxi Province, China
Source: PLoS One. 2014 Oct 6;9(10):e109476. doi: 10.1371/journal.pone.0109476 (PMC4186885; doi:10.1371/journal.pone.0109476)
Supplement: Tables S3 — The significant association between different levels of CDC staff and their perception towards infectious disease surveillance. (DOCX) [file pone.0109476.s003.docx]

**Table S3.** The significant association between different levels of CDC staff and their perception towards infectious disease surveillance

| **Variables** | | **Perception towards surveilliance of infectious disease** | | | | | ***p*** |
| --- | --- | --- | --- | --- | --- | --- | --- |
|  |  | **EW** | **VW** | **JS** | **B** | **Total** |  |
| Levels of CDC | |  |  |  |  |  |  |
|  | Provincial | 7 | 28 | 21 | 3 | 59 |  |
|  | Prefecture-level city | 55 | 124 | 27 | 4 | 210 | 0.001 |
|  | District/county | 6 | 30 | 9 | 0 | 45 |  |
|  | Total | 68 | 182 | 57 | 7 | 314 |  |
| Professional level | |  |  |  |  |  |  |
|  | Junior | 22 | 82 | 23 | 0 | 127 |  |
|  | Intermediate | 31 | 74 | 17 | 5 | 127 | 0.020 |
|  | Senior | 15 | 26 | 17 | 2 | 60 |  |
|  | Total | 68 | 182 | 57 | 7 | 314 |  |

Note: EW= Extremely well; VT = Very well; JS = Just so so; B = Bad.
