# Supplementary material for: The Impact of Climate Change on Infectious Disease Transmission: Perceptions of CDC Health Professionals in Shanxi Province, China
Source: PLoS One. 2014 Oct 6;9(10):e109476. doi: 10.1371/journal.pone.0109476 (PMC4186885; doi:10.1371/journal.pone.0109476)
Supplement: Table S4 — Local CDC staff's perceptions towards the response to infectious disease epidemics (N = 314). (DOCX) [file pone.0109476.s004.docx]

**Table S4.** Local CDC staff’s perceptions towards the response to infectious disease epidemics (N=314)

| **Variable** | **ET** |  | **VT** |  | **JS** |  | **B** |
| --- | --- | --- | --- | --- | --- | --- | --- |
|  | **n (%)** |  | **n (%)** |  | **n (%)** |  | **n (%)** |
| Response to food-borne disease | 163 (51.9) |  | 130 (41.4) |  | 18 ( 5.7) |  | 3 (1.0) |
| Response to water-borne disease | 149 (47.5) |  | 126 (40.1) |  | 33 (10.5) |  | 6 (1.9) |
| Response to vector-borne disease | 134 (42.7) |  | 137 (43.6) |  | 34 (10.8) |  | 9 (2.9) |
| Response to air-borne disease | 184 (58.6) |  | 108 (34.4) |  | 18 ( 5.7) |  | 4 (1.3) |
| Response to others | 134 (42.7) |  | 127 (40.4) |  | 49 (15.6) |  | 4 (1.3) |

Note: ET= Extremely timely; VT = Very timely; JS = Just so so; B = Bad.
